# Barriers to pre-treatment genomic characterization of the small renal mass

**DOI:** 10.18632/oncotarget.26756

**Published:** 2019-03-01

**Authors:** Jamil S. Syed, Cedric Lebacle, Brian Shuch

**Affiliations:** Department of Urology, Institute of Urologic Oncology (IUO), David Geffen School of Medicine at UCLA, Los Angeles, CA, USA

**Keywords:** RCC, genomics, biopsy, kidney cancer, active surveillance

## Small renal mass clinical challenge

The widespread availability of cross-sectional imaging has led to a rise in incidentally detected small renal masses (≤ 4 cm). Stage 1 tumors are now found in 60-70% of all new kidney cancer diagnoses [1, 2]. Most small renal tumors will remain indolent after detection and it has been consistently shown that the vast majority are not destined for metastatic spread. With a slow growth rate and low potential for stage progression in the small renal mass, the role of active surveillance for these tumors has expanded. The median age of diagnosis for kidney cancer is 64 years and many of these patients have significant competitive comorbidities. Surgery may expose these patients to possible unnecessary treatment or overtreatment for benign and indolent tumors [3]. As such, the American Urologic Association recommends active surveillance for small renal masses detected in the elderly and infirm patients. Although active surveillance is an adequate option for certain patient populations, it is not a guarantee that treatment will be avoided indefinitely as patients may undergo delayed treatment due to tumor growth kinetics or even patient anxiety. As it remains, there are no universally accepted guidelines for determining the need for upfront or delayed intervention in the small renal mass. Thus, there remains a need to better identify patients at highest risk in hopes of avoiding unnecessary over-treatment and maximizing survival.

## Issues with molecular profiling in kidney cancer

Renal cell carcinoma represents a genetically diverse population of cancers. Despite our knowledge of adverse pathologic features which portend to worse survival in the small renal mass such as nuclear grade [4], histology alone may not fully characterize the risk of progression and metastasis. With the introduction of clinical cancer genetics in the realm of kidney cancer, there has been an opportunity to correlate altered genomic events and expression patterns to progression and survival after surgical resection. For patients with small renal masses, molecular profiling with a percutaneous biopsy represents a modality that can be potentially utilized for stratifying disease risk prior to treatment. A perceived obstacle to this is the concern for tumor heterogeneity. As opposed to a large renal mass where multiple biopsies can be easily obtained for adequate characterization of subclonal events, the small renal mass may only be able to accommodate a single biopsy and there is concern that clinically meaningful driver alterations may go undetected given tumor heterogeneity.

## A homogenous tumor profile

To address the issue of genomic heterogeneity in the small renal mass (SRMs) Ueno et al. compared 23 small (< 4 cm) and 24 large (> 7 cm) renal masses [5]. The study is the largest of its type to date *(n* = 47) and analyzed three ≥ 1 cm apart designated regions from each tumor. Genomic heterogeneity was assessed through 3 methods: DNA copy number variation (CNV), and mRNA gene expression by 2 transcriptomic RNA classifiers: the clear-cell A and B system and the CCP Score. From CNV, small renal masses were found to have significantly less subclonal events when compared to large tumors. On gene expression profiling, 4.7% (1/21) *vs.* 23.8% (5/21) had mixed clear-cell A and B classifications in the SRMs and large masses, respectively. While median CCP scores did not differ between the SRMs and large masses, there was an increased variance found for large masses.

## Implications to management

The findings demonstrate that SRMs had limited copy number variation and transcriptomic heterogeneity. The homogeneous expression profile would theoretically strengthen the argument that an enhanced renal mass biopsy, a standard biopsy plus incorporation of genomic profiling, may be useful for patients with a small renal mass as the limitations of tumor heterogeneity may not be as pronounced as they are in larger tumors. The incorporation of such a tool clinically has the potential to shift the diagnostic paradigm for patients with SRMs and help decide who may benefit most from early intervention. While this approach is now technically feasible, high cost and the lack of clinical utility studies may prevent widespread adoption. Our center is currently developing assays to lower the cost of assessment and evaluating the clinical utility in the management of the SRM.
